# Dr John Hurst MB, DPM, FRCPsych: Formerly Consultant in Psychiatry of Intellectual Disability, Lea Castle Centre, Wolverly, Nr. Kidderminster, Worcestershire

**DOI:** 10.1192/pb.bp.115.052001

**Published:** 2016-04

**Authors:** Meera Roy, Ashok Roy

**Figure:**
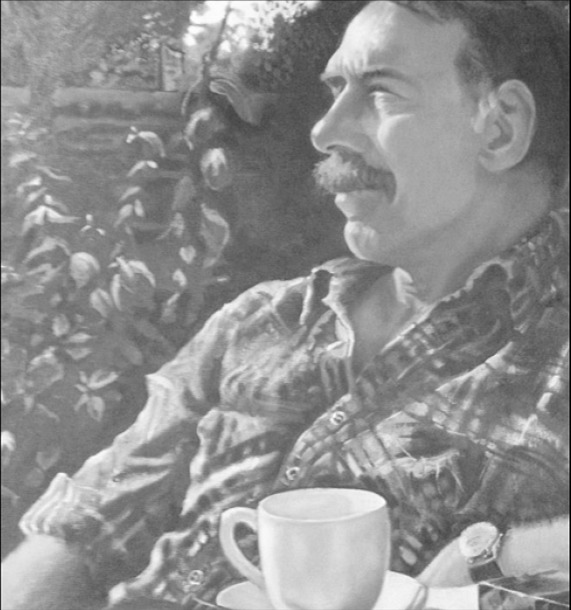


Dr John Hurst's approach to the treatment of people with intellectual disabilities was ahead of its time. In the early 1970s, John, who has died aged 79, developed the pre-discharge unit at South Ockenden Hospital, Essex, as a therapeutic community, as well as a number of community group homes. He also introduced one of the earliest community nursing services in intellectual disability there between 1972 and 1975. Between 1974 and 1977, as Chair of the Outward Housing Group in Waltham Forest, he was instrumental in bringing together housing associations, Mencap and community nursing to provide supported group homes for former in-patients. Through the Clifford Trust at Leytonstone Hospital, he was involved in fostering work with children with profound disability who were placed in institutions and used music therapy to increase their awareness and communication. He moved to Lea Castle Hospital in 1978, initially as the consultant responsible for the deaf/blind unit, where he introduced the teaching of Makaton signing for people with severe intellectual disabilities and deafness.

In 1982 – following an incident in which a 20-year-old woman with mild intellectual disability had to spend 6 months in custody because the courts could find no suitable placement for her – John became the consultant to a newly created unit for people with mild intellectual disabilities and mental illness, implementing therapeutic community principles to meet patients' emotional needs. He was a pioneer in the use of psychotherapy for people with intellectual disabilities and published on the benefits of holiday homes for people with intellectual disabilities and their families. He was pivotal in the setting up of community teams to support people with intellectual disabilities in Telford and Malvern during his time as community consultant there, preferring to work directly with people with intellectual disabilities and their carers, rather than take the wider managerial role often expected of National Health Service (NHS) consultants.

He was a member of the mental health charity MIND working party on intellectual disabilities in 1977 and gave evidence to the Royal Commission on the NHS about services for people with intellectual disabilities. He was nominated to serve on the Mental Health Act Commission and was active in the development of the Code of Practice of the Mental Health Act 1983. John was a member of the Board of Examiners for the Royal College of Psychiatrists for several years and was actively involved in teaching psychiatric trainees in the Birmingham rotation. A generation of consultants in intellectual disability in the West Midlands have fond memories of not only being trained by him, both as registrars and senior registrars, but also of being fed delicious meals at his home during their placement with him.

John was born in London on 16 September 1935 but his family moved to Oxfordshire where he grew up with a sister with an intellectual disability. He and his family were closely associated with the growth of the Oxfordshire Spastics Society and its facilities. He went to Kidlington Infants School and remembered knitting balaclava helmets for the forces during the Second World War. He attended Bicester Grammar School and qualified in medicine from Birmingham Medical School in 1960. He undertook training in psychiatry in Birmingham, obtaining the Diploma in Psychological Medicine (DPM) in 1968. He undertook further training in the psychiatry of intellectual disability in Birmingham and was appointed consultant psychiatrist in South Ockenden Hospital, Essex, in 1970. He was a Foundation Member of the Royal College of Psychiatrists and became a Fellow in 1986. He was the first author of a paper published in 1994 describing a specialist unit for people with intellectual disabilities and mental illness.^1^

John lived with his long-term partner Ian and their home was a haven of peace for stressed ex-trainees and friends. He and Ian entered into a civil partnership in 2006 at a touching ceremony at the ‘Plough and Harrow’ in Birmingham. John had a lifelong passion for classical music and opera and used to entertain his friends' children telling them how he used to listen at the door of churches in Oxford as a child. After his retirement in 1991, he and Ian travelled extensively, putting pins on the world map in their conservatory to mark the many places they had visited.

In the summer of 2011, John became ill with septicaemia secondary to cellulitis around a foot ulcer which he developed on a cruise to Turkey, just before the world cruise he and Ian had planned. He spent 3 months in hospital, sadly developing cognitive changes. He was always a gentle, loving person and even as he stopped being able to do things for himself, his warm personality shone through. He had several hospital admissions in his last year and whenever we visited him, we were always greeted with a delightful smile. He slipped away in his sleep on 9 October 2014 after he had had a cup of coffee and a chat with his physiotherapist. The nurse in charge said that telephoning Ian that day was the most difficult telephone call she had to make in all her life.

John was a wonderful and caring clinician, teacher and friend who could find something good in everyone and never said a harsh word about others. He leaves behind his partner Ian and his extended family of ex-trainees and their children, all of whom miss him greatly.
